# From fragility to resilience: A systems approach to strengthen primary health care

**DOI:** 10.3389/fpubh.2022.1073617

**Published:** 2023-01-09

**Authors:** Elizabeth Lugten, Rachel Marcus, Rhea Bright, Farzana Maruf, Nazo Kureshy

**Affiliations:** ^1^Credence Management Solutions, Vienna, VA, United States; ^2^USAID Bureau for Global Health, Office of Health Systems, United States Agency for International Development, Washington, DC, United States; ^3^Social Solutions International, North Bethesda, MD, United States; ^4^Global Health Technical Assistance and Mission Support Project, Washington, DC, United States

**Keywords:** primary health care (PHC), health security, essential public health functions, health system strengthening (HSS), resilience

## Introduction

Twenty years of progress in service coverage has been estimated to be neutralized by the COVID-19 pandemic (1). 2022 marks the second consecutive year that the world has not progressed toward the 2030 Sustainable Development Goals (SDGs) due to multiple and often concurrent health and security crises (2). COVID-19 exposed the fragility of health systems in some of the wealthiest countries and demonstrated how inequities within and across countries are compounded by public health threats. Public health threats expand beyond disease outbreaks, to include climate change, conflict, and other shocks to the health system. Regardless of the threat, the fact remains that both sudden and slow-onset disturbances will happen and health systems need to be adequately prepared to mitigate disruptions to health care. Strengthening primary health care (PHC) systems is critical for bolstering countries' health systems' ability to effectively respond to and recover from new and recurring shocks, while preventing backsliding of health outcomes (3, 4). For purposes of this paper PHC is defined as a “*whole-of-society approach to health that aims at ensuring the highest possible level of health and well-being and their equitable distribution through comprehensive integrated health services that embrace primary care as well as public health goods and functions, supported by multi-sectoral policies and actions that address the social determinants of health and engage and empower individuals, families and communities*” (5, 6).

As reinforced in the 2018 Astana Declaration commitments (7), PHC and systems that support and facilitate it are central to strengthening and connecting UHC, health system resilience and pandemic preparedness within countries (8); this has been increasingly recognized in global efforts to improve preparedness and response to health emergencies (9). IA strong PHC platform with existing community trust can help support response efforts through early diagnosis and reduced demand on hospitals through accessibility (10). For example, in Indonesia climate change is increasing flooding, droughts, and erosion, in turn increasing health problems due to impacts on water quality, access to health facilities or increase in infectious diseases. Community based risk management programs supported PHC systems in communities facing changing patterns of vector and waterborne diseases. With only 8 years remaining to achieve the SDGs, continued setbacks in improving equitable access and affordability of quality essential health services threaten to make universal health coverage (UHC) unattainable by 2030.

We must remember that people are at the heart of health care, and resilient, quality health systems are responsive to patient and population needs. Effective PHC can address 80% of a person's health needs by providing promotive, preventive, curative and rehabilitative services accordingly (11). An additional $200–370 billion (USD) a year is needed to scale PHC in low and middle income countries; WHO recommends that countries allocate or reallocate 1% of their GDP to PHC from government and external funding sources to help close this gap (5). Increasing funding levels to strengthen integrated PHC systems is necessary for bolstering health system resilience capacities, which can reduce the need to divert health funds away from essential routine services during times of crisis due to shocks or stressors. While increasing funding for PHC is important, it is not enough. Greater attention needs to be directed to methods that help understand the context-specific barriers to high performance of integrated PHC, including effective integration of essential public health functions (EPHFs) and integration across disease-specific inputs, so that it is more responsive and comprehensive for all people at every life stage, especially marginalized and vulnerable populations.

## Discussion

Understanding how a system functions, including barriers to performance during each stage of shock, can provide a clear roadmap for improving health system resilience and strengthening high-quality integrated, people-centered services.^1^ A systems approach uses systems thinking (15) to understand how health system components function, evolve, behave, and interact, enabling the identification of barriers to achieving equity, quality, and resource optimization (16). This discussion provides examples of how using a systems approach can strengthen PHC and increase health system resilience.

### Effective integration of essential public health functions in primary health care systems

Developing flexible health systems that can make shifts to respond to shocks, yet maintain essential health services, requires strategic integration of PHC and EPHFs (17, 18). EPHFs (such as monitoring of health status, supporting efficient and effective multi-sectoral planning and preparedness, disease surveillance and response, and advancing public health research) (19) are the minimum requirements and capacities for systems to ensure public health, and are recognized as key for health system resilience (20). Strong PHC systems that integrate EPHFs are better positioned to proactively detect shocks and respond to surge support needs. Thus, systems with integrated PHC and EPHFs can improve health security and resilience during crisis and recovery (5). Although it is recognized that EPHFs are an important part of PHC, there are few examples of countries that have integrated them well (18). Analyses that use a systems approach can help illuminate opportunities and pathways for investment in PHC integration with EPHFs, as structures and approaches differ across country and region (21). PHC should be strengthened to enable countries to rapidly adapt and transform both health system functions and public health functions to ensure adequate availability of human, financial and supply resources when and where they are needed the most before, during, and after crises.

### Reducing fragmentation

Creating resilient health systems also requires a global shift away from predominantly siloed disease-specific inputs, which can limit countries' ability to effectively respond to shocks, and toward a balanced approach with integrated health system investments as well. Program experience has repeatedly shown the value of strengthening alignment and collaboration among country governments, non-governmental partners, donors, and multilateral institutions, in strengthening PHC and improving community trust (22). Countries are increasingly focusing reforms for high performing primary health care at the community level by applying a systems lens. For example, funding for community health worker programs has been historically heavily fragmented, and analyses in sub-Saharan Africa show that taking a strong systems approach is necessary to be impactful to reduce fragmentation and “establish mechanisms for accountability to encourage harmonization of donor funding”. Investments toward building sustainable national CHW program delivery models should be embedded within the PHC system in order to be an efficient and effective use of funds and support better quality of care (23). Fifteen countries are engaging with the Community Health Roadmap partnership (24), an innovative collaboration between governments, funders, and partners, to advance national policy and systems priorities to accelerate progress toward health outcomes. Estimates indicate the lives of up to 2.4 million women, children, and newborns could be saved each year “if a complete package of evidence-based interventions was provided - and accessible - at the community level (25)”. Examples of emerging priorities for institutionalizing community health that require urgent action across the fifteen countries include professionalizing the community health workforce, developing data systems, and engaging communities to build local governance and accountability (24). A key cross-country priority is strengthening and sustaining CHW compensation, often by addressing political and financial challenges to institutionalizing CHW payment (26).

### Improving equity and accountability

Systems approaches to strengthening people-centered PHC offer opportunities to engage in equity-oriented research and practice that can transform the health system, address power imbalances, and recognize key equity drivers within complex systems (27). Health inequities and health system shocks continue to disproportionately affect the most vulnerable, with 50% of the world's population lacking access to essential health and social services and 100 million people being pushed into poverty annually from paying for health care out-of-pocket (22). This is especially true in environments affected by protracted conflict, which often lack the requisite human, financial, and supply resources for basic PHC. Approximately 24% of the world's population, about 1.8 billion people, live in fragile contexts where delivering quality essential health services is challenged (28). Community engagement and multisectoral systems-based approaches are key to strengthening health system weaknesses to ensure continuity of essential services, increase flexibility during response and recovery, and addressing underlying social determinants of health. Evidence shows that PHC designed in an inclusive manner that integrates EPHFsbest addresses the broad range of health needs that individuals, their families, and communities require (29, 30).

PHC that does not meet the needs of users—due to issues such as insufficient funding levels for optimal system performance leading to health worker, medicine, equipment, and commodity shortages; lower quality care and accountability; inequitable access; and/or required out-of-pocket payments—can shift care-seeking behavior away from PHC providers to higher levels of specialized care (31, 32). Engaging relevant civil society and community based groups in governance of PHC at all levels creates more opportunity for accountability and reduces fragmentation of services (33). Building social accountability structures and increasing community ownership and engagement in planning, prioritization and delivery of PHC services can reduce the asymmetry of power between health system actors such as policy makers and providers and individuals accessing care (34, 35). In turn, social accountability can increase the provision of respectful care, which can improve health outcomes in communities through improved trust leading to more use of the healthcare system, minimized medication adherence issues, and even improve working conditions of health workers by reducing burnout (36). At the national level, policy dialogue efforts that are well-resourced, clear and collaborative can enable participants to effectively engage in the process (37) and can lead to high levels of policy commitment (38).

### Supporting system-wide process improvement

While access is important to ensuring equity, quality of care drives utilization. Health care services must be safe, effective, and person-centered. More deaths in low- and middle-income (LMIC) countries occur as a result of poor-quality care than from lack of access (35). Systems-practice includes incorporating principles of process improvement to continually assess and address identified gaps in health system performance. Country-led efforts to build absorptive, adaptive, and transformative capacities to mitigate the impact of shocks and stressors is an example of system-wide process improvement. As the context changes over time, processes within the health system need to be established, modified, or terminated in order to maintain optimal performance (39). For example, some LMICs were unsure of how best to include the private sector in planning for initial national response efforts to COVID-19 (40), exposing a systems process gap. In most LMICs, governments have focused on delivering public sector services themselves rather than establishing governing mechanisms and processes that integrate the public and private sectors in a mixed health system. National health sector planning should consider intentional and strategic linkages with the private sector to strengthen PHC to improve quality of care and expand access to services, especially as part of crisis responses.

### Measuring impacts and learning

Finally, system-wide learning and measurement are also key to quality health system responses to a shock (41). Research and learning should inform longer term system transformation and improvement in policy and practice to support recovery and preparation for the next shock. For example, systematic analysis of bottlenecks to strengthen community health systems as part of PHC revitalization efforts in West and Central Africa identified a range of health system barriers related to health financing, essential medical technology and products, and integrated health service delivery, but only some of these barriers had been self-identified by participating countries. The systems analysis, which utilized a community health system bottleneck analysis tool, was critical to identifying the full range of opportunities to strengthen PHC (42). Further, though health systems collect data on health system inputs, such as workforce and logistics, countries often lack data about performance and processes used to improve equitable quality care (33). Incorporating measurement of performance-related indicators or perception of quality of care can fill knowledge gaps to inform decision-making and can be used to strengthen political will to strengthen PHC platforms.

## Conclusion

Systems approaches to strengthening people-centered PHC can enable context-specific understanding of health system needs and opportunities, and therefore can be an inclusive, effective and efficient approach to enhancing health and wellbeing and maintaining health system resilience and health security during crisis and recovery. Strong political leadership and commitment to engaging with stakeholders that impact health and its determinants at all levels and to adapting health systems to the social and economic contexts is needed to successfully develop and sustain resilient, integrated PHC systems and reduce inequities (32). Systems practice facilitates a whole-of-society approach to the investments needed for stronger health systems that can meet the needs of everyone, especially marginalized and vulnerable populations. As countries take stock of their roadmaps or commitments toward progress for UHC in 2023, reinvigorating PHC based on stakeholder engagement and alignment in support of country-led PHC platforms and longer-term health system goals will be essential for achieving the SDGs and mitigating adverse health consequences during future crises.

## Author contributions

EL conceptualized and led the development of the commentary. RM, RB, FM, and NK provided technical inputs and made key contributions to the work. All authors reviewed the commentary. All authors contributed to the article and approved the submitted version.

## Footnotes

^1^See, e.g.: (12–14).
